# Highly permeable PVDF membrane with PS/ZnO nanocomposite incorporated for distillation process

**DOI:** 10.1039/c8ra02908c

**Published:** 2018-06-27

**Authors:** Ramin Roshani, Fatemeh Ardeshiri, Majid Peyravi, Mohsen Jahanshahi

**Affiliations:** School of Chemical Engineering, Kavosh Institute of Higher Education Mahmood Abad Iran; Nanotechnology Research Institute, Babol Noshirvani University of Technology Shariati Ave. Babol 47148-71167 Iran mmohse@yahoo.com http://www.nano.nit.ac.ir +98 1132320342 +98 1132320342; Institute of Nanoscience and Nanotechnology, University of Kashan P.O. Box: 87317-53153 Kashan Iran

## Abstract

In order to enhance the flux and wetting resistance of PVDF membranes for MD applications, we have developed a novel PVDF blend nanocomposite membrane using a polystyrene/ZnO (PS/ZnO) hybrid nanocomposite. The PS/ZnO nanocomposite was synthesized by free radical polymerization of styrene in the presence of vinyltrimethoxysilane (VTMS) grafted on the surface of ZnO nanoparticles. The blend nanocomposite membrane is fabricated *via* the phase inversion method and we examined the effects of the PS/ZnO nanocomposite on porosity, mechanical properties, hydrophobicity, LEPw, morphology, surface roughness and MD performance. It was found that the addition of the PS/ZnO hybrid nanocomposite (0.25, 0.5 and 0.75%) resulted in an increase in porosity (>70%), which is attributed to increased pore size and reduction of the spongy layer thickness. Furthermore, the addition of the nanocomposite also improved the surface roughness and contact angle. Comparison between the neat and modified membrane shows that with incorporation of the PS/ZnO nanocomposite, the desalination flux of 30 g L^−1^ saline aqueous solution significantly increased and rejection reached 99.99%. Meanwhile, during 100 hours continuous desalination process, the membranes composed of 0.75% PS/ZnO hybrid nanocomposite exhibited high performance stability (15.79 kg m^−2^ h^−1^) compared with the neat PVDF membrane.

## Abstract 2

In order to enhance the flux and wetting resistance of PVDF membranes for MD applications, we have developed a novel PVDF blend nanocomposite membrane using a polystyrene/ZnO (PS/ZnO) hybrid nanocomposite.

## Graphical abstract

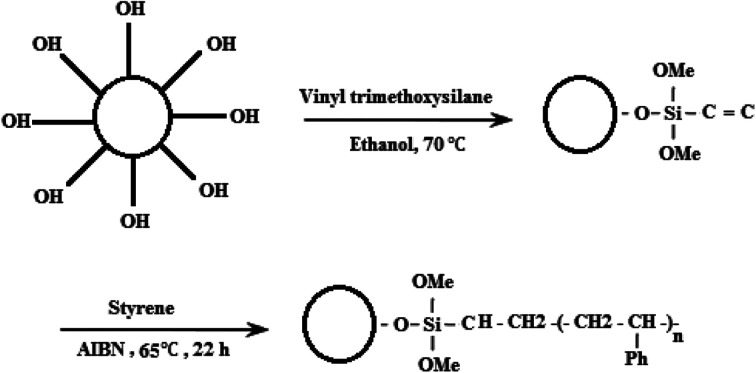

## 1. Introduction

Owing to its intrinsic advantages, such as low operating temperature, low hydrostatic pressure, low operating temperature, waste heat and renewable energy sources (solar or geothermal sources), and eventually low energy consumption, membrane distillation (MD) is considered as a preferred and practical approach to saline water desalination and is able to compete with pressure-based membrane processes.^1–3^ Nevertheless, from the commercial standpoint, this process has not been actualized yet in the industry, mainly owing to high operation costs, low flux and, most importantly, wetting phenomena.^4^ MD is a thermal membrane separation process in which a hydrophobic microporous membrane acts as a barrier between the hot feed solution and cold distillate stream, across which the partial vapor pressure gradient creates a driving force to penetrate water vapor molecules from the feed to the permeate side.^5,6^ In order to improve flux and prevent pore wetting, a membrane should have some particular features, such as superhydrophobicity and high liquid entry pressure (LEP), as well as high porosity (70–80%) together with higher pore size.^7,8^ Typically, three types of commercial microporous polymeric membranes have been the subjects of many investigations; these are polyvinylidenefluoride (PVDF), polytetrafluoroethylene, and polypropylene.^9,10^ In the present work, we focused on the latter presented limitation and developed a modified PVDF membrane because PVDF as a common base membrane material has been widely used in MD processes owing to its accessibility and appropriate solubility at room temperature in common organic solvents.^11^

It is known that MD membrane performance is dependent on hydrophobicity, surface chemistry and structural characteristics, such as thickness, porosity and pore size.^12^ In recent years, many attempts have been made to create superhydrophobic surfaces, of which several approaches can be mentioned: sol–gel method, electrospinning, etching, layer by layer-assembly, low-temperature hydrothermal, and deposition. However, application of some of these approaches to fabricate superhydrophobic surfaces is restricted owing to high costs and complex operating conditions.^13–15^

The blending of nano-additives into PVDF polymeric membranes can be executable as an achievable, facile, affordable, and practical strategy.^16,17^ Generally, there are two ways to increase the hydrophobicity of inorganic nanoparticles. One is performed through surface absorption or reaction with small molecules, such as alkyl and fluorosilane compositions, and has been widely used for MD applications.^18^ For instance, Razmjou and co-workers^19^ prepared a superhydrophobic membrane by TiO_2_ fluorosilanization using the low surface energy material *H*,1*H*,2*H*,2*H*-perfluorododecyltrichlorosilane. They examined the anti-fouling performance of modified membranes in a DCMD process. Hong *et al.*^20^ improved hydrophobicity of PVDF membranes by depositing fluorographite particles on the membrane surface. Another is based on the coating of a hydrophobic polymer chain onto the surface of nanoparticles (polymeric nanocomposite) that has not been applied to MD applications.^20^ From another point of view, in some studies, nano-additives are incorporated into MD micro-porous polymeric membranes to obtain narrow pore size distribution, high roughness and to increase LEP. Hou and co-workers^21^ examined the effects of calcium carbonate nanoparticles on the permeability of MD membranes. The nanoparticles optimized the surface roughness, enlarged the pores size, improved porosity and increased permeate flux without an occurrence of pore wetting. Tijing *et al.*^22^ fabricated PVDF-carbon nanotube electrospun membranes and found that the presence of nanoparticles increased the contact angle and surface roughness.

Nanoscale ZnO as a multifunctional inorganic nanoparticle has been widely applied in the membrane modification process. As reported by Hong *et al.*,^23^ the porosity of a PVDF microfiltration membrane was enhanced by addition of nano ZnO particles. They found that the mechanical and thermal strengths of the modified PVDF membrane increased considerably, which are known as vital properties for the MD process. Chen *et al.*^24^ used modified ZnO nanoparticle for fabricating omniphobic membranes for direct contact membrane distillation owing to the richest growth morphologies. According to the reasons mentioned above, ZnO nanoparticles can improve the structural properties of PVDF membranes along with hydrophobicity enhancement by polymerizing styrene segments on ZnO nanoparticles. Styrene polymer was chosen based on its intrinsic properties and because PS is an aromatic hydrocarbon polymer made from the monomer styrene, which is well known as a low surface energy material that has been widely used to prepare electrospun superhydrophobic surfaces for applications such as the MD process.^25,26^ However, the literature is not yet focused on nanoparticles modified with PS for MD applications. The present study describes the development of a new PVDF blend nanocomposite membrane using a PS/ZnO hybrid nanocomposite as an additive and the investigation of its MD performance using 30 g L^−1^ saline aqueous solution. The PS/ZnO hybrid nanocomposite is synthesized by free radical polymerization to graft PS chains on vinyltrimethoxysilane (VTMS)-treated ZnO nanoparticles. In this study, we tried to examine not only the synthesis and morphology of the PS/ZnO nanocomposite by Fourier-transform infrared spectroscopy (FTIR) and scanning electron microscopy (SEM), but also the effects of the nanocomposite on the morphology, porosity, mechanical properties and especially MD performance by the addition of different concentrations of PS/ZnO nanocomposite into the PVDF casting solution.

## 2. Materials & methods

### 2.1. Materials

To prepare MD membranes, polyvinylidene fluoride polymer (PVDF, Koreha Company) was used as the membrane base polymer and *N*,*N*-dimethylformamide (DMF, >99.8%, Merck) as the solvent. Materials used for the synthesis and modification of ZnO nanoparticles including zinc nitrate hexahydrate, sodium carbonate, vinyltrimethoxysilane (VTMS), ethanol (99.8%), styrene (St) and the initiator 2,2-azobis (isobutyronitrile) (AIBN) were supplied by Merck. The performance of the MD membranes was examined using sodium chloride (NaCl, >99%, Dr Mojallali) solution. Distilled water was used throughout this study.

### 2.2. Synthesis of ZnO nanoparticles

ZnO nanoparticles were prepared through precipitation method and calcination.^27^ Briefly, zinc nitrate hexahydrate and sodium carbonate were dissolved in distilled water at concentrations of 0.1 and 0.12 M, respectively. Then, zinc nitrate hexahydrate aqueous solution was slowly added to sodium carbonate aqueous solution under vigorous stirring until forming a milky sol. The obtained suspension solution was filtered and then washed three times with distilled water. The resulting nanoparticles were dried at 60 °C for 24 h and then calcined at 250 °C for 2 h.

### 2.3. Modification of ZnO nanoparticles with VTMS

To prepare VTMS-grafted ZnO, 2 g of the synthesized ZnO nanoparticles and 4 ml of VTMS were dispersed in 100 ml of ethanol then the mixed solution was refluxed and stirred at 70 °C overnight. After centrifugation of the obtained product, the VTMS-grafted nanoparticles were washed with ethanol and then dried under vacuum at 50 °C for 24 h.

### 2.4. Preparation of PS/ZnO nanocomposite

The free radical polymerization was conducted in a three-necked round-bottom flask (100 ml) equipped with a condenser and nitrogen bubbler, which was placed in an oil bath. 1.5 g of the grafted ZnO nanoparticles was dispersed in 100 ml of ethanol for 15 min. Then, 3 g of St monomer and 0.1 g of AIBN initiator were added to the suspension. After bubbling with nitrogen for 30 min, the mixture was heated at 65 °C for 22 h under stirring. After polymerization, the reaction mixture was centrifuged and washed three times with ethanol. The product was purified by toluene (25 °C) three times and then dried at 50 °C under vacuum for 24 h.^28^

### 2.5. Preparation of PVDF nanocomposite membrane

The developed membranes were prepared by phase inversion method.^29^ The casting solution was composed of PVDF powder, DMF and different concentrations of modified ZnO. The compositions of the casting solutions are listed in Table 1. For the preparation of the casting solution, all components were gradually added to DMF and mixed by stirring for 24 h at 25 °C. When the mixing of the dope solutions was completed, they were degassed at ambient temperature for about 3 h to release the trapped air bubbles. The dope solution was cast on non-woven fabric polyester using a casting knife with a thickness of 120 μm. After being exposed for 30 s in air, the membranes were smoothly immersed in a non-solvent bath (water). The flat-sheet membranes were stored in the water bath for at least 1 day to ensure to remove the excess solvent. At the end, the membranes were dried on filter paper for 24 h at room temperature.

**Table 1:** Compositions of casting solutions

Membrane	PVDF (%)	Modified ZnO (%)	DMF (%)
Pure PVDF	16	0	84
0.25% PS/ZnO/PVDF	15.75	0.25	84
0.5% PS/ZnO/PVDF	15.5	0.5	84
0.75% PS/ZnO/PVDF	15.25	0.75	84

### 2.6. Membrane characterization

#### 2.6.1. Structural characterization assay

The chemical compositions of the bare and modified ZnO nanoparticles were investigated using a Fourier transform infrared spectroscopy (FT-IR) (model WQF-520, Germany). The morphological structure of the ZnO NPs and the polystyrene–ZnO hybrid nanocomposites was determined by scanning electron microscopy (SEM, Seron, AIS2100, South Korea). Energy-dispersive X-ray spectroscopy (EDX) was applied to chemically characterize the prepared nanoparticles. In order to characterize the contact angle, suspensions of ZnO and PS/ZnO hybrid nanocomposite were firstly cast onto glass substrate and then dried at room temperature.

Field emission scanning electron microscopy (FESEM, model: Mira 3-XMU) was used to examine the surface and cross-section morphology of the membranes. The surfaces roughness of the membranes was evaluated using atomic force microscopy (AFM, model: Easyscan2 flex). The three-dimensional structure of the membrane surface was observed in a scan area of 10 μm × 10 μm. The average roughness was computed by SPM DME analysis software and was employed to examine the morphology of the membranes. The tensile strength was determined using a tensile test machine (Instron 5966) at a crosshead speed of 5 mm min^−1^ with an initial length of 20 cm. Five specimens were tested for each hollow fiber sample and the average of the five specimens was calculated. Contact angle measurements were taken at three random locations of each sample to minimize the experimental error and then a reliable value was reported.

#### 2.6.2. Liquid entry pressure of water (LEPw) measurements

LEPw measurement was conducted using a dead-end filtration cell according to the procedure presented by Smolder and Franken.^30^ The feed chamber of the dead-end filtration cell was filled with distilled water and connected to a nitrogen gas cylinder. First, the membranes were kept at constant pressure (about 0.25 bar) for at least 10 minutes. Then, the pressure was slowly increased in small steps (0.25 bar) every 10 min until the first permeate drop was obtained at the cell outlet; the corresponding pressure was reordered as the LEPw value.

#### 2.6.3. Membrane porosity and tortuosity

To determine the membrane porosity, the gravimetric method was used.^31^ The membranes were cut into 1 cm × 1 cm pieces and immersed in ethanol for 30 min. After mopping with tissue paper, their weights were measured as wet weight. The membrane porosity (*ε*) was obtained using eqn (1):1
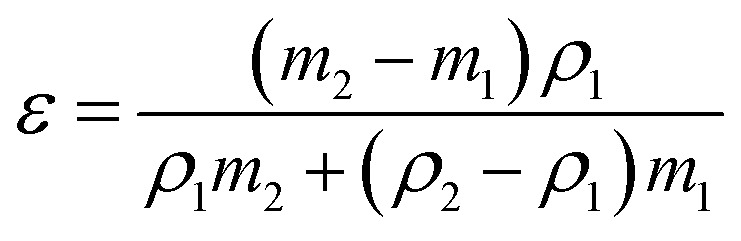
where *m*_1_ is the dry weight and *m*_2_ is the wet weight of the membranes, and *ρ*_1_ and *ρ*_2_ are the density of PVDF (1.78 g cm^–3^) and ethanol (0.789 g cm^–3^), respectively.

Tortuosity (*τ*) has an inverse relationship with porosity and could be calculated by the following formula, using the membrane porosity:^32^2
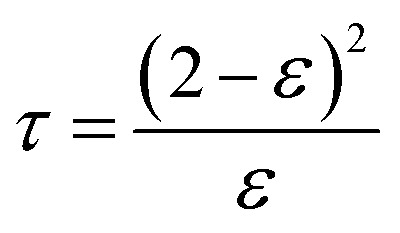


#### 2.6.4. Experimental procedure

The flux and rejection of the bare and modified membranes were evaluated by DCMD experiments. The membranes with 30.4 cm^2^ effective area were positioned in a membrane plate module, which consisted of hot feed and permeate sections. The 30 g L^−1^ saline solution was used in the hot feed at a temperature and flow rate of 70 °C and 400 ml min^−1^, respectively. Also, the distilled water is employed in the permeate and was kept at a constant temperature of 22 °C using an ice bath. The rate flow of the permeate was adjusted to 200 ml min^−1^. The flow rate was measured by rotameter. To measure the permeate mass of the MD membranes, the distillate tank solution was placed on an electronic analytical balance. The whole experiment was carried out under the mentioned conditions during the MD test. The water flux (kg m^−2^ h^−1^) was calculated using eqn (3):3
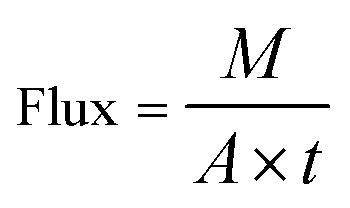
where *M* (kg) is the amount of permeate collected per unit area of effective membrane, *A* (m^2^) is the effective membrane surface area, and *t* (h) is operation time of flux collection. Furthermore, the rejection was calculated according to following formula:4
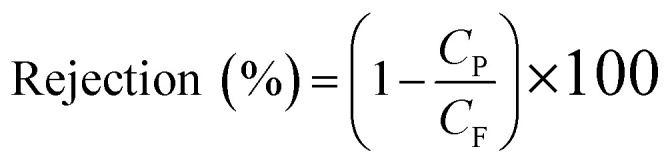
here *C*_P_ and *C*_F_ are the concentrations of the permeate and feed solutions, respectively. Salt concentrations of the aqueous NaCl solutions were determined *via* a conductivity meter (model: WA-2017SD, Taiwan).

## 3. Results and discussion

### 3.1. PS/ZnO hybrid nanoparticle

**Fig. 1:**
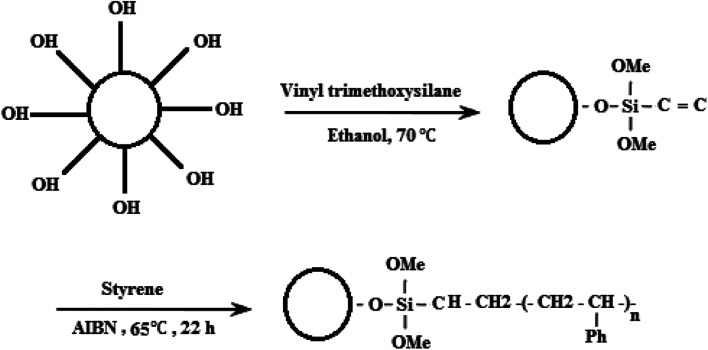
Surface modification of ZnO nanoparticles with VTMS and PS.

There are two challenges in the synthesis of polymeric nanocomposites: (i) nanoparticles tend to agglomerate in the polymer matrix, which leads to poor performance of the composite,^32^ and (ii) ZnO is an inorganic material while styrene is an organic material, so their compatibility is not very good.^33^ One approach to solve this problem has been the grafting of the hydrophobic polymer chain onto the surface of the nanoparticles by covalently bonding to the hydroxyl groups present on the nanoparticles.^34^ In fact, the properties of the polymer-grafted nanoparticles can be tailored through proper selection of the species used for the grafting monomers and the grafting conditions. During the synthesis of ZnO nanoparticles through precipitation method, the use of zinc nitrate hexahydrate as a main precursor creates hydroxyl groups upon the ZnO nanoparticle for the next modification step. Therefore, the silane coupling agent is used to graft polymer on the ZnO surface. The silane coupling agent has an active end to react with the hydroxyl groups on the surface of the ZnO nanoparticles and one end with a vinyl group for performing radical polymerization. Fig. 1 indicates the chemical reaction of VTMS with ZnO nanoparticles and free radical polymerization of St monomer for formation of the polymeric shell on the ZnO nanoparticle surface. The synthesized ZnO nanoparticles were grafted with VTMS by a reaction between the hydroxyl groups of the ZnO nanoparticles and the methoxy groups of VTMS. This leads to grafting VTMS with the free head of the carbon–carbon double bond on the ZnO nanoparticle as an inorganic core. The polymeric shell on the ZnO nanoparticle surface was accomplished *via* free radical polymerization through copolymerization of St monomer with the vinyl group of VTMS on the core surface, which leads to the formation of the polystyrene–ZnO hybrid nanocomposite.^26^

**Fig. 2:**
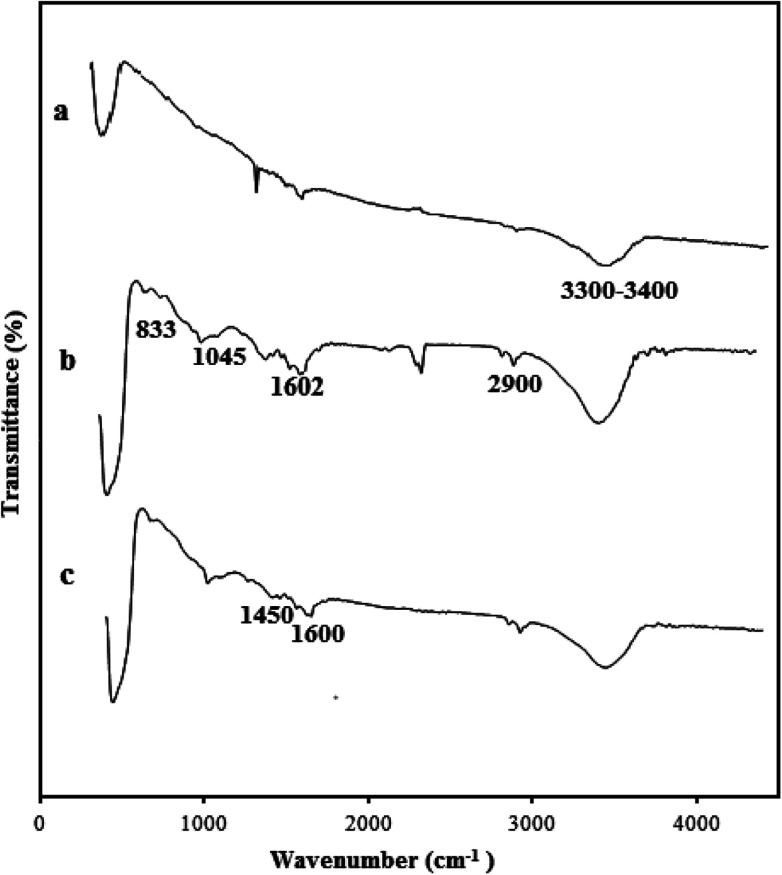
ATR-IR spectra of (a) ZnO nanoparticles, (b) ZnO-grafted VTMS and (c) PS/ZnO hybrid nanocomposite.

IR spectroscopy was used to confirm the interaction of ZnO nanoparticles with VTMS and PS. The FT-IR spectra of bare ZnO (a), VTMS-grafted ZnO (b) and the PS/ZnO nanocomposite (c) are shown in Fig. 2. The broad peak between 3300 cm^−1^ and 3400 cm^−1^ corresponds to hydroxyl groups of the ZnO nanoparticles.^35^ The peaks at 1045, 833, 3300 and 2900 cm^−1^ are attributed to Si–O, Zn–O–Si and methylene groups (CH_2_) on the VTMS-grafted ZnO surfaces, respectively. The peaks located at 1602 cm^−1^ can be assigned to the C=C double bond of VTMS before reaction with (Fig. 2b). The vibration peaks at 1450 and 1600 cm^−1^ correspond to the C=C stretching in aromatic rings of PS (Fig. 2c).^36^ As shown in Fig. 2c, the intense multi-vibration bands around 3000 and 767 cm^−1^ belong to the C–H groups of aromatic rings, and are increased by grafting PS on the VTMS-grafted ZnO surface.^37^ Consequently, all the observed data show that the VTMS and PS have been successfully attached on the ZnO nanoparticle surface.

**Fig. 3:**
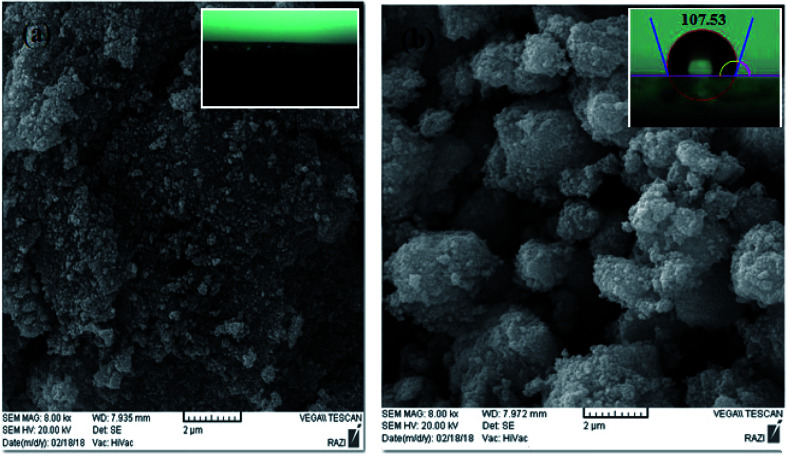
The morphology of ZnO nanoparticles (a) and ZnO/PS hybrid composite (b).

The representative SEM images of ZnO and the PS/ZnO hybrid nanocomposite are shown in Fig. 3, which indicates the surface morphology. The magnified surface morphology of unmodified ZnO exhibited a quasi-spherical shape with particle size of about 20–30 nm. It is observed that the PS/ZnO hybrid nanocomposite was more spherical in comparison with the ZnO nanoparticles. The interaction of styrene monomer with the V-ZnO nanoparticles *via* polymerization is the reason for the high spherical shape of the PS/ZnO hybrid nanocomposite. The inset images in Fig. 3 show the contact angle measurements. The results indicate that ZnO nanoparticles make the surface hydrophilic in terms of a contact angle of 0° for water. Owing to the strong affinity of the electron donor sites of ZnO to interact with the hydroxyl groups of water molecules, the water droplets dispersed on the surface. The water contact angles of the VTMS-ZnO and PS/ZnO nanoparticles were 83.60° and 107.53°, respectively. The modified ZnO coating with PS exhibits a superhydrophobic surface owing to the presence of hydrophobic groups, *i.e.* the benzene of PS, that can repel water droplets from the surface.

**Fig. 4:**
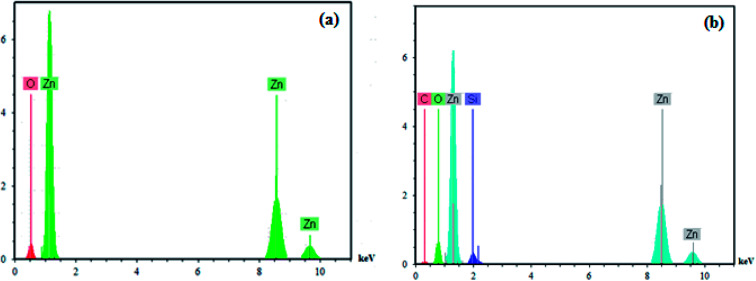
EDX analysis of ZnO (a) and PS/ZnO hybrid composite (b).

Additionally, the presence of elements in the ZnO nanoparticles and PS/ZnO hybrid nanocomposite was analyzed from their EDX spectra. As observed in Fig. 4a, the EDX spectrum of the ZnO nanoparticles showed the presence of Zn and O elements. In PS/ZnO hybrid nanocomposite (Fig. 4b.), the existence of C and Si elements in addition to Zn and O elements was detected using the strong peaks in EDX.

### 3.2. Morphological study of PS/ZnO-PVDF membrane

**Fig. 5:**
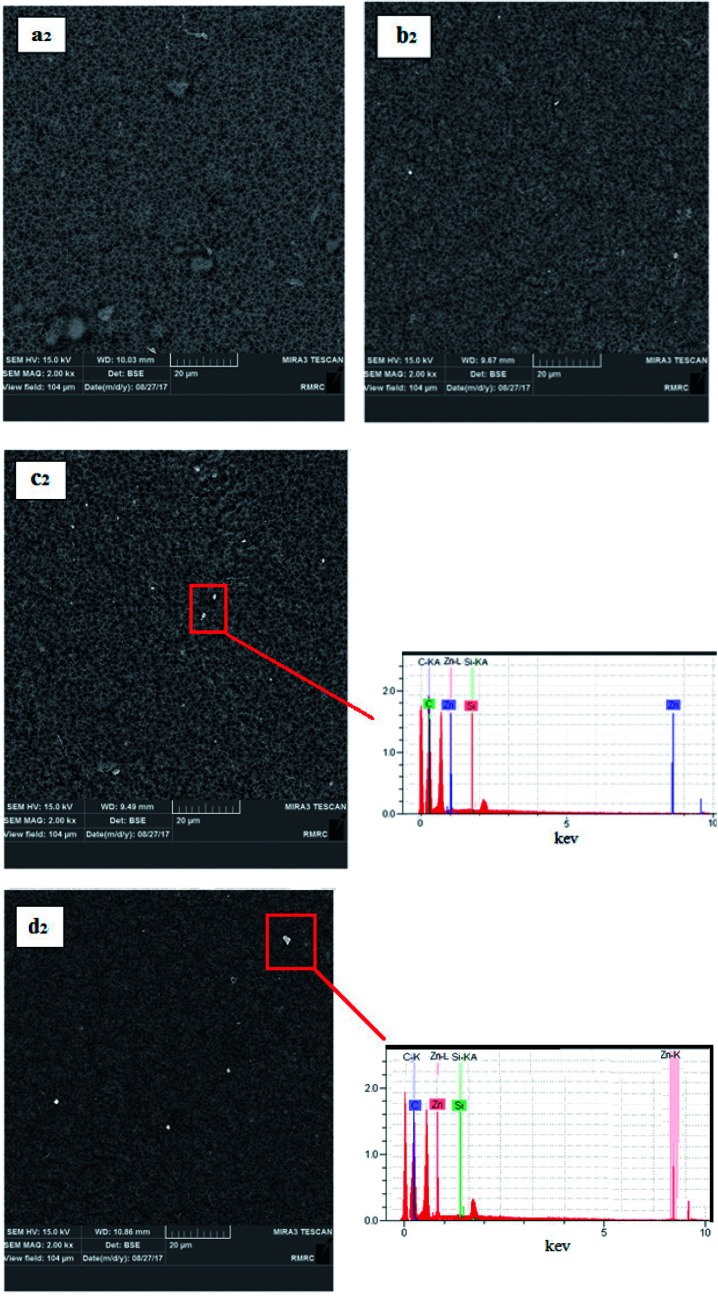
Membrane surface images: Pure PVDF (a2), 0.25% modified ZnO/PVDF (b2), 0.5% modified ZnO/PVDF (c2) and 0.75% modified ZnO/PVDF (d2).

Fig. 5 shows the images of the top surface of the pure PVDF membrane and the PS/ZnO nanocomposite membrane. A comparison between the surface images of the bare PVDF and PS/ZnO-PVDF membranes indicates that nanoparticles (white spots) were uniformly dispersed on membrane surface. Moreover, it should be pointed out that incorporation of the PS/ZnO nanocomposite into casting solution did not lead to agglomeration with loadings of 0.25 and 0.5%, although agglomeration was partially observed with loading of 0.75% PS/ZnO nanocomposite. The existence of polymer brushes, *i.e.* PS, in the core–shell nanocomposite structure led to poor interaction with PS/ZnO nanocomposite, repelling from each other and reducing agglomeration.^38^ In order to confirm that the white spots are agglomerated nanoparticles, EDX analyses of 0.5% and 0.75% modified ZnO/PVDF were carried out on the surface containing white spots (Fig. 5). The presence of Zn and Si peaks in the EDX spectra indicates that white spots can be considered as a region including nanoparticles. From these SEM surface images, it seems that a few particles were distributed over the membrane surface. This case can be ascribed to the nature of the involved components in the casting solution including water (as nonsolvent), modified nanoparticles and PVDF polymer. Regarding the hydrophilicity of the non-solvent and the hydrophobicity of the modified ZnO nanoparticles, the nanoparticles did not tend to migrate to the membrane top surface. In other words, the nanoparticles strongly interacted with PVDF polymer chains owing to hydrophobic interactions and as a result fixed into the inner structure of the membrane.

**Fig. 6:**
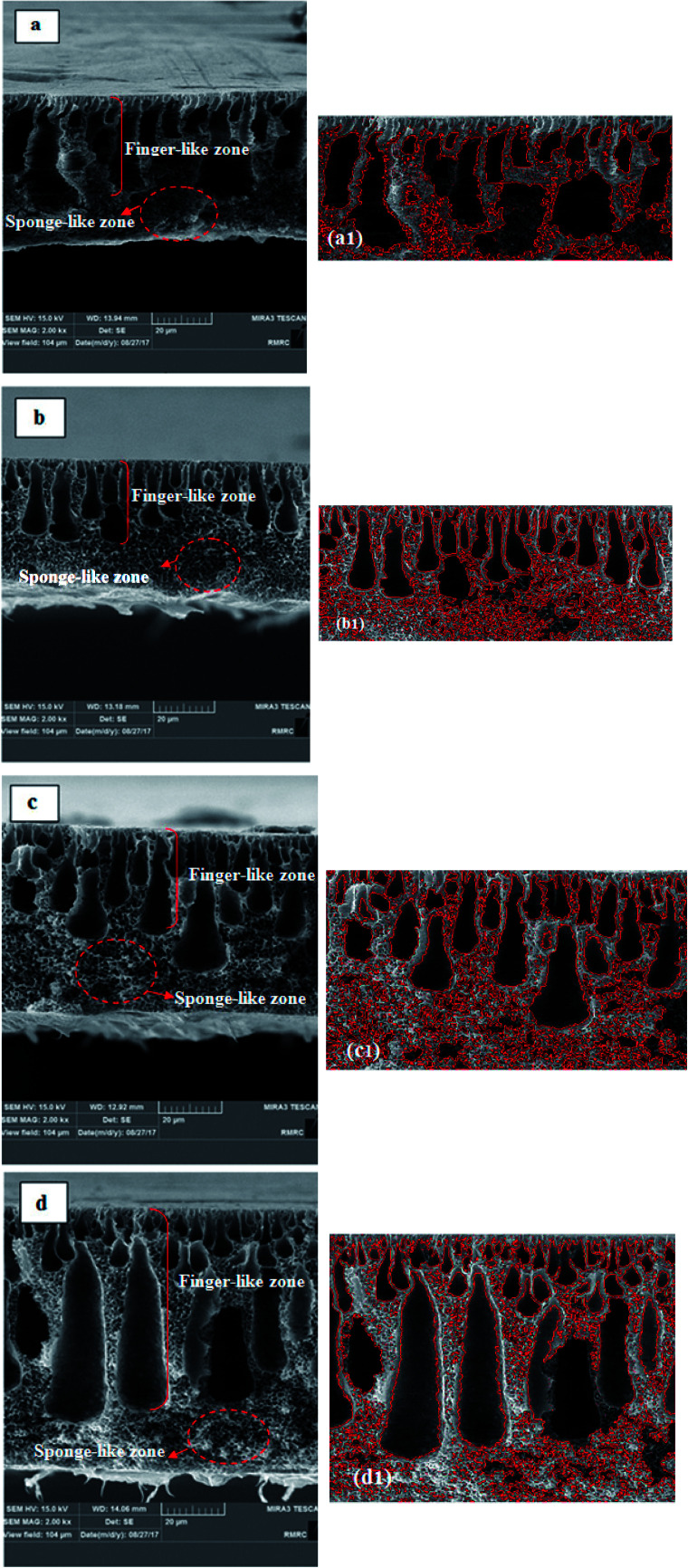
Cross-section images and the analyzed SEM images of (a and a1) pure PVDF membrane, (b and b1) 0.25% modified ZnO/PVDF membrane, (c and c1) 0.5% modified ZnO/PVDF membrane and (d and d1) 0.75% modified ZnO/PVDF membrane.

Fig. 6 shows the cross-section images of the prepared membranes. From these SEM images, it can be clearly seen that all the membranes were composed of two zones: finger-like and sponge-like. With the addition of 0.25% hydrophobic PS/ZnO nanocomposite into the casting solution, the sponge-like structure became more porous and it was full of very small spherical nodules. The thickness of the top skin layer was reduced in comparison with the pure PVDF membrane. The finger-like macrovoids changed to tear shapes with wider ends. It can also be obviously seen that the size of the finger-like macrovoids is looser and more regular than those of the pure membrane. In the case of the 0.5% modified ZnO/PVDF membrane; it seems that the thickness of the sponge layer was reduced slightly compared to that in the 0.25% modified ZnO/PVDF membrane. In comparison with the pure and 0.25% modified ZnO/PVDF membranes, the size of the finger-like macrovoids looked irregular and the top surface was more porous. For the 0.75% modified ZnO/PVDF membrane, the top layer of the finger-like zone was composed of small cavities so there seemed to be many more of them than in the other three membranes. It can also be observed that the finger-like macro void was long and narrow with uniform size. In general, with the addition of the hydrophobic PS/ZnO nanocomposite into the casting solution, the size of the finger-like macro void increased and the length of top layer became longer and more porous, which led to increased porosity and would enhance the permeability of the MD membranes. On the other hand, in the pure PVDF membrane the spherical nodules and sponge-like structure could not easily be observed, but with addition of nanoparticle from 0.25%, the sponge-like structure began to appear in the bottom and between the finger-like macro voids. However, when the concentration increased to 0.75%, the thickness of the sponge-like layer decreased. The differences observed between the morphology of the pure and modified PVDF membrane could be attributed to the combined interaction between components (nanoparticle and polymer) and thermodynamic properties. In order to determine the membrane porosity from the cross-sectional FESEM image quantitatively, an image analyzer^39^ was applied and the analyzed images are shown in Fig. 6. The image analyzer used MATLAB programming to convert the qualitative observation of SEM images to quantitative data. The mean of the void equivalent diameter of the membrane was calculated using this software. The analyzed SEM images indicated that the pure PVDF membrane has a dense sponge structure in which the number of spherical nodules is lower than in the other membranes. It is clear that with the addition of the nanocomposite into the casting solution, a sponge-like structure with small spherical nodules appears between the macrovoids of the finger-like zone and that the thickness of this structure reduces in the 0.75% PS/ZnO/PVDF membrane. Moreover, it can be observed that the number and size of macrovoids of the nanocomposite membrane in the finger-like zone is increased by loading the hydrophobic nanocomposite into the casting solution. The mean void equivalent diameter calculated by the SEM analyzer software for the pure, 0.25, 0.5 and 0.75% PS/ZnO/PVDF membranes were 0.3476, 0.4429, 0.4467 and 0.4613 μm, respectively. The calculations show that the mean of the void size increased significantly by increasing the amount of nanocomposite up to 0.75 wt%. According to the results obtained from the SEM analyzer software, the authors believe that blending of modified ZnO nanoparticles produces a drastic change in the morphology of the nanocomposite membranes in comparison with the pure PVDF membrane.

In many nanocomposite membranes, when nanoparticles are added into casting solution, the nanoparticles act as a nucleation factor and accelerate the phase separation rate, consequently leading to the formation of big macro voids in the sub layer and enhancement of the top layer thickness. In many polymeric nanocomposite membranes, when inorganic nanoparticles are employed, depending on the amount of nanoparticles, they can act as nucleating and anti-nucleating agents. Hou and co-workers^21^ reported that when an appropriate quantity of CaCO_3_ nanoparticles was employed in a PVDF membrane (0–3%), the exchange rate between solvent and non-solvent was accelerated, reducing the thermodynamic stability of the dope and consequently resulting in the formation of finger-like structures with large pores. However, by adding nanoparticles in excess (4.5–7%) into the casting solution, the nanoparticles act as anti-nucleating agents, so the formation of multiple nuclei suppresses the formation of macrovoids by allowing additional nuclei to form rapidly in front of prior formed nuclei, preventing existing nuclei from expanding into macrovoids to form more spherical morphologies. Similar results were observed with alumina,^40^ zeolite^41^ and titanium oxide.^42^ As a result, suppression of macrovoids and creation of spherical morphologies was only observed at high nanoparticle loadings while modified nanoparticles with low concentration were applied in this study. In this regard, the nucleating agent provides additional sites and reduces the activation energy for nucleus formation.^43^ The addition of proper amounts of nucleating agent into the casting solution increases the chance of diffusing into the polymer lean phase and could easily help to increase the formation rate of nuclei, which ultimately leads to larger finger-like macrovoids.^44^

As previously mentioned, there is the poor interaction between nanocomposite structures owing to the presence of the PS shell on the ZnO nanoparticles. Thereby, on addition of the nanocomposite into the casting solution, the nanocomposite has the strong tendency to interact with polymer matrix. This resulted in the good compatibility of the components with the DMF solvent, which can penetrate into the polymer-lean phase area, and increased solvent and nonsolvent exchange. Consequently, this gives another explanation for the pore size enhancement with nanocomposite addition.^45,46^

**Fig. 7:**
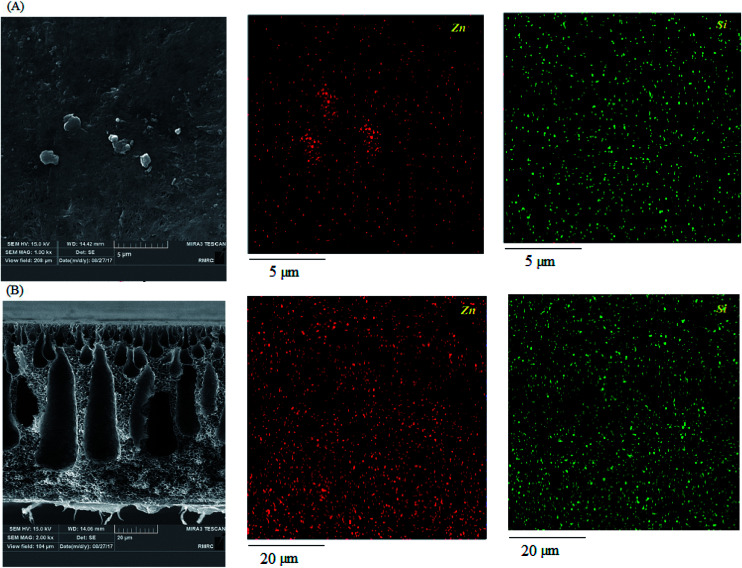
EDX mapping of the surface (A) and cross-section (B) of the nanocomposite membrane containing 0.75% nanoparticle loading.

To clarify the presence of the nanoparticles, EDX mapping images of the cross section and surface of 0.75% modified ZnO/PVDF were obtained and are presented in Fig. 7. The images clearly show the uniform dispersion of the hydrophobic ZnO nanoparticles across the membrane surface and structure. It is evident that Zn and Si mapping corresponded to ZnO and the coupling agent, respectively. It also confirms the presence of the coupling agent upon the ZnO nanoparticles from reaction with the hydroxyl groups of nanoparticles and provides active support for the polymerization of styrene monomer upon the ZnO nanoparticles.

**Fig. 8:**
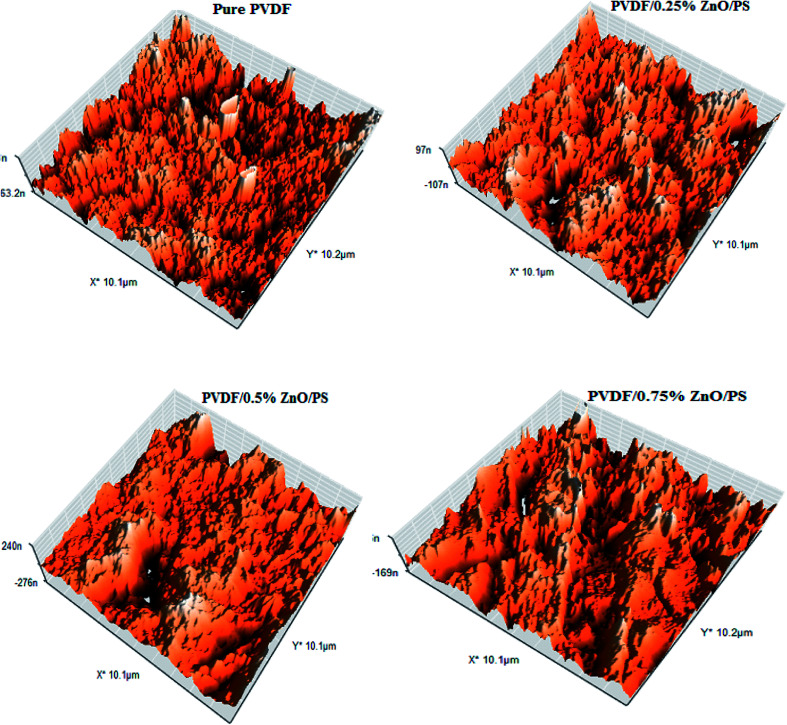
Three-dimensional surface AFM images of the pure and modified PVDF membranes.

The three-dimensional surface AFM images of the bare and modified PVDF membranes at a scan size of 10 μm × 10 μm are presented in Fig. 8. It can be seen from these images that the PVDF membranes containing different amounts of nanocomposite generally presented a rougher surface as compared with the bare PVDF membrane. This was confirmed by the hills and valleys structure visually seen on the surface of the modified PVDF membranes.

**Fig. 9:**
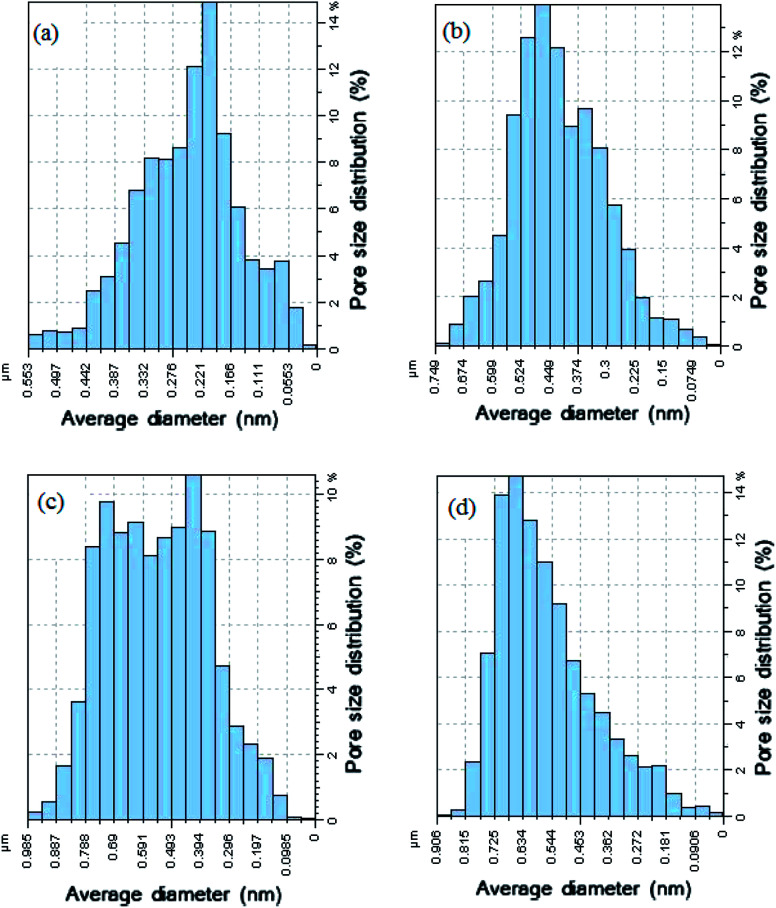
Pore size distributions of PVDF membranes prepared with: (a) 0 wt% PS/ZnO, (b) 0.25 wt% PS/ZnO, (c) 0.5% wt% PS/ZnO and (d) 0.75% wt% PS/ZnO.

The membrane contact angles and surface roughness parameters obtained from AFM images using SPM DME software are summarized in Table 2. When the nanoparticles were added into the polymer matrix, the *S*_a_ value sharply decreased from 26.13 nm for the bare membrane to 20.20 nm for the PVDF membrane containing 0.5% modified ZnO. By increasing the modified ZnO content to 0.75%, the *S*_a_ (the mean roughness) value was significantly increased to 58.79 nm. This can be explained by the morphological changes during the phase inversion. In addition, the pore size of the membranes is an effective agent of surface roughness based on the reviewed literature^47,48^. Regarding the roughness parameter's dependence on the *Z* value, when the membrane surface consists of deep depressions (large pores), the piezoelectric scanner tip moves up and down over a wide range. Since the vertical distance of large pores is higher than in comparison with shallow depressions, the roughness parameter increased.^49,50^ This usually happens in a porous membrane consisting of large surface pores. To clarify this issue more, we calculated the pore size distributions from AFM images using the Nanosurf Report presented in Fig. 9. The results indicated that the average pore size of the PVDF membranes increased after loading PS/ZnO nanoparticles (0.25, 0.5 and 0.75 wt%). As shown in Fig. 9, the pore size distributions were shifted to the large pore diameters by increasing the PS/ZnO concentration in the casting solution. Based on this supposition and the presence of nanoparticles on the membrane surface, the results showed higher roughness as compared with the pure PVDF membrane. The obtained contact angle results were in accordance with the surface roughness results, so that the highest contact angle showed the highest surface roughness.^51^ The presence of hydrophobic groups, *i.e.* the benzene of PS, in the hybrid nanocomposite structure can be considered as another reason for the contact angle enhancement. Moreover, the embedment of the nanocomposite into the PVDF matrix not only increases the surface roughness and contact angle but also can contribute to MD performance improvement by increasing the surface area contact with vapor molecules, which reduces mass transport resistance.^52,53^ Although PS has low surface energy, it did not enhance the membrane hydrophobicity significantly. The contact angle can be influenced by the surface properties of the membrane, including porosity and pore diameter.^54^ According to the Laplace–Young equation, because of the capillary force, the water drops could gradually penetrate into the porous surface and the larger pores indicate lower resistance against water penetration, which led to the decrease of the contact angle.^55^ The increased porosity and pore size distributions with the addition of the nanoparticles into the casting solution influenced the contact angle results. As a result, the long chain PS acts as a hydrophobic group and tends to increase the contact angle while the increased porosity decreases the contact angle.

**Table 2:** Surface roughness parameters and contact angles of pure and modified PVDF membranes

Membrane	Roughness			Contact angle
	*S*_a_	*S*_q_	*S*_z_	
Pure PVDF	20.20	28.59	44.71	73
0.25% PS/ZnO/PVDF	29.27	37.27	297.03	89
0.5% PS/ZnO/PVDF	58.79	77.23	788.59	99
0.75% PS/ZnO/PVDF	47.19	59.89	576.79	92

### 3.3. Mechanical properties

**Fig. 10:**
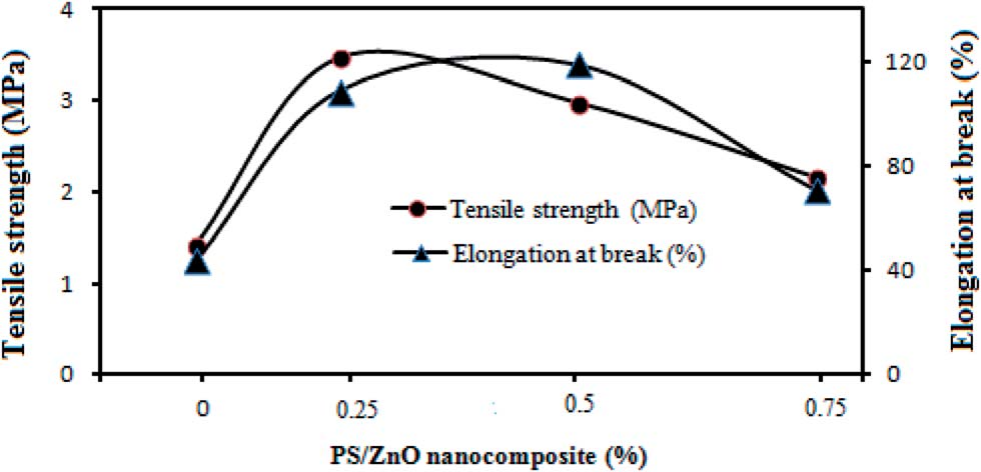
Mechanical strength of the PVDF nanocomposite membranes with various % of PS/ZnO nanocomposite content.

Although the MD process, unlike pressure-driven membrane processes, is usually operated without hydraulic pressure, investigation of the mechanical properties of MD membranes is required for long term operation owing to the high operation temperature.^22,56^Fig. 10 shows the effect of the PS/ZnO hybrid nanocomposite on the mechanical strength of the nanocomposite membrane. It can be seen that the PVDF membranes containing the PS/ZnO hybrid nanocomposite showed higher elongation at break and tensile strength in comparison with the neat PVDF membrane. With the addition of the PS/ZnO nanocomposite content into the casting solution, the elongation at break increased from 44% for the neat PVDF membrane to 119% for the 0.5% PS/ZnO nanocomposite membrane and indicated a significant increase in tensile strength, then decreased to 71% for the 0.75% PS/ZnO nanocomposite membrane. Tensile strength increased from 1.42 MPa for the neat PVDF membrane to 3.47 MPa for the 0.25% PS/ZnO nanocomposite membrane, and then decreased to 2.16 as the PS/ZnO nanocomposite content was further increased. These changes might be interpreted through morphological results. The FESEM images indicated that the addition of this nanoparticle greatly influenced the membrane structures. It can alter the mechanical properties of the composite membranes. Although all membranes had a sponge-like cross-section, some morphological differences were presented in their FESEM images. When a low concentration of the PS/ZnO nanocomposite (0.25 and 5%) was incorporated within the membrane structure, the thickness of the sponge-like structure was higher in comparison with that in the 0.75% PS/ZnO nanocomposite membrane. Based on the reviewed literature, membranes with a sponge-like morphology have superior mechanical properties.^57,58^ The high mechanical strength can be attributed to the cross-linker behavior of this nanoparticle that is attained by the dispersion of inorganic nanoparticle into the polymeric matrix.^59,60^ In the case of the cross-linker behavior of these nanoparticles, it suggested that the presence of PS/ZnO hybrid nanocomposite within the membrane matrix limits the movement of the PVDF polymeric chains segments and increases the rigidity of the polymeric chains. Indeed, the nanoparticles can act as temporary crosslinks between the polymer chains, which provides localized regions of enhanced strength. Consequently, to break down the bond between this nanoparticle and the PVDF chains would require a lot of energy.^61,62^ Therefore, both the elongation at break and tensile strength of the nanocomposite membranes (0.25 and 0.5% PS/ZnO nanocomposite) were improved.

The decrease of the mechanical strength in the 0.75% PS/ZnO nanocomposite membrane can be ascribed to two reasons: (i) the presence of large pores in the sub-layer^63^ and (ii) the limitation of free movement of polymer chains with the excessive loading of the nanocomposite into the casting solution.^64^

### 3.4. Structural assay of PS/ZnO-PVDF membrane

The changes in the structure of the bare PVDF membrane by adding PS/ZnO nanocomposite at different concentrations are presented in Table 3. The porosity of the modified membranes with PS increased while the tortuosity decreased in the presence of PS/ZnO nanocomposite in the membrane scaffold. The results show that the porosity of the 0.25% modified ZnO/PVDF membrane is doubled in comparison with that of the pure PVDF membrane. This can be attributed to morphology difference. The analyzed SEM images and morphology for the a1 and b1 membranes indicates that the a1 membrane possesses expanded macrovoids in a smaller number. However, the existence of many small and uniform macrovoids in the b1 membrane cross section causes the higher porosity. On the other hand, a1 exhibited a finger-like structure while the cross-sectional structure of the 0.25% PS/ZnO nanocomposite membrane in b1 can be divided into two zones: the finger-like upper zone and the sponge-like bottom layer that shows spongy pores distributed almost over the entire bottom layer of the cross-section. As reported by Zhao,^65^ the sponge-like structure owing to the high density of pores is more favorable than the finger-like structure for the improvement of membrane porosity for MD performance.

**Table 3:** The porosity, tortuosity and LEP of the PVDF membranes with different modified ZnO concentrations

Membrane	Porosity (%) of PS/ZnO/PVDF	Tortuosity of PS/ZnO/PVDF	LEP (bar) of PS/ZnO/PVDF	Porosity (%) of VTMS/ZnO/PVDF without PS
Pure PVDF	38 ± 0.97	5.1 ± 0.97	8 ± 1.0	38 ± 0.97
0.25% modified ZnO/PVDF	65 ± 0.83	2.5 ± 0.83	6.25 ± 0.6	61 ± 0.4
0.5% modified ZnO/PVDF	68 ± 0.93	2.3 ± 0.93	6.1 ± 0.8	62 ± 0.4
0.75% modified ZnO/PVDF	73 ± 0.85	2.07 ± 0.85	5.8 ± 0.2	64 ± 0.5

Among all the prepared membranes, the PVDF membrane containing 0.75% PS/ZnO nanocomposite showed the most porosity, which is desirable for high MD performance since it provides more surfaces for vapor molecules to pass through membrane pores, leading to increased flux.^66^ It is worth mentioning that we measured the porosity of the nanocomposite membrane modified by ZnO-VTMS without PS by the gravimetric method three times. As shown in Table 3, the porosity of the membrane modified by ZnO-VTMS without PS was significantly lower than that of the membrane with PS. This can be attributed to the short chains of VTMS that are not able to interact with the polymer matrix leading to weak compatibility of the components with the DMF solvent and decreased solvent and nonsolvent exchange. Consequently, this case resulted in porosity reduction with VTMS-ZnO addition.^45,46^

The decreasing LEP from 8 bar for pure the PVDF membrane to 5 bar for the 0.75% of PS/ZnO nanocomposite was mainly because of the presence of large pores, as discussed for the membrane morphology. However, there were no significant changes to the LEP values of the modified membrane on increasing the PS/ZnO nanocomposite from 0% to 0.75%. Despite the decline in the LEP values of the modified membranes, the obtained results were still acceptable for achieving high membrane performance for MD application and preventing saline aqueous solution penetration through the dry pores.

### 3.5. The effects of temperature on the flux

**Fig. 11:**
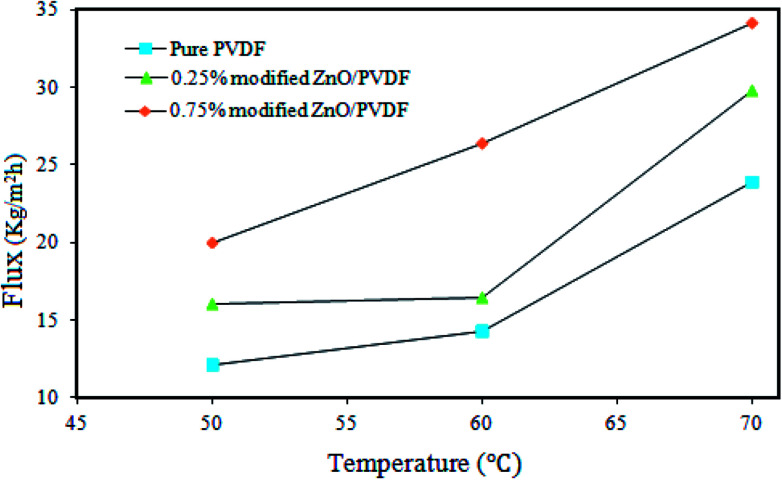
Effects of temperature on the DCMD performance of the pure PVDF membrane and the modified membrane PS/ZnO hybrid nanocomposite (0.25% and 0.75%).

The performance of the pure PVDF, 0.25% and 0.75% PS/ZnO membranes was investigated at 50, 60 and 70 °C using 30 g L^−1^ NaCl aqueous solution as the feed in a DCMD process (Fig. 11). All membranes were tested under the same operating conditions. It was seen that the permeate flux significantly increased by increasing the temperature of hot feed solution from 50 to 70 °C for each corresponding membrane. During the experiments, no salt leakage through the membrane in the cold distillate streams was observed even at high temperature; rejection was 99.99%. This predictable result is attributed to the acceptable fact of the MD process.^67,68^ In other words, when the temperature of the hot feed solution is increased, the water vapor pressure gradient increases at the interface between the hot feed solution and distillate, eventually resulting in the generation of high permeate flux. Furthermore, another conclusion found from Fig. 11 was that with the addition of the polystyrene–ZnO hybrid nanocomposite in the casting solution, the flux of the permeates simultaneously increased with the increment of the hot feed solution temperature. The highest flux was obtained for the membrane containing 0.75% polystyrene–ZnO hybrid nanocomposite. Based on the obtained results, we conducted performance tests on the membranes at 70 °C in the MD process using 30 g L^−1^ NaCl aqueous solution.

### 3.6. Effects of polystyrene–ZnO hybrid nanocomposite on the DCMD performance

**Fig. 12:**
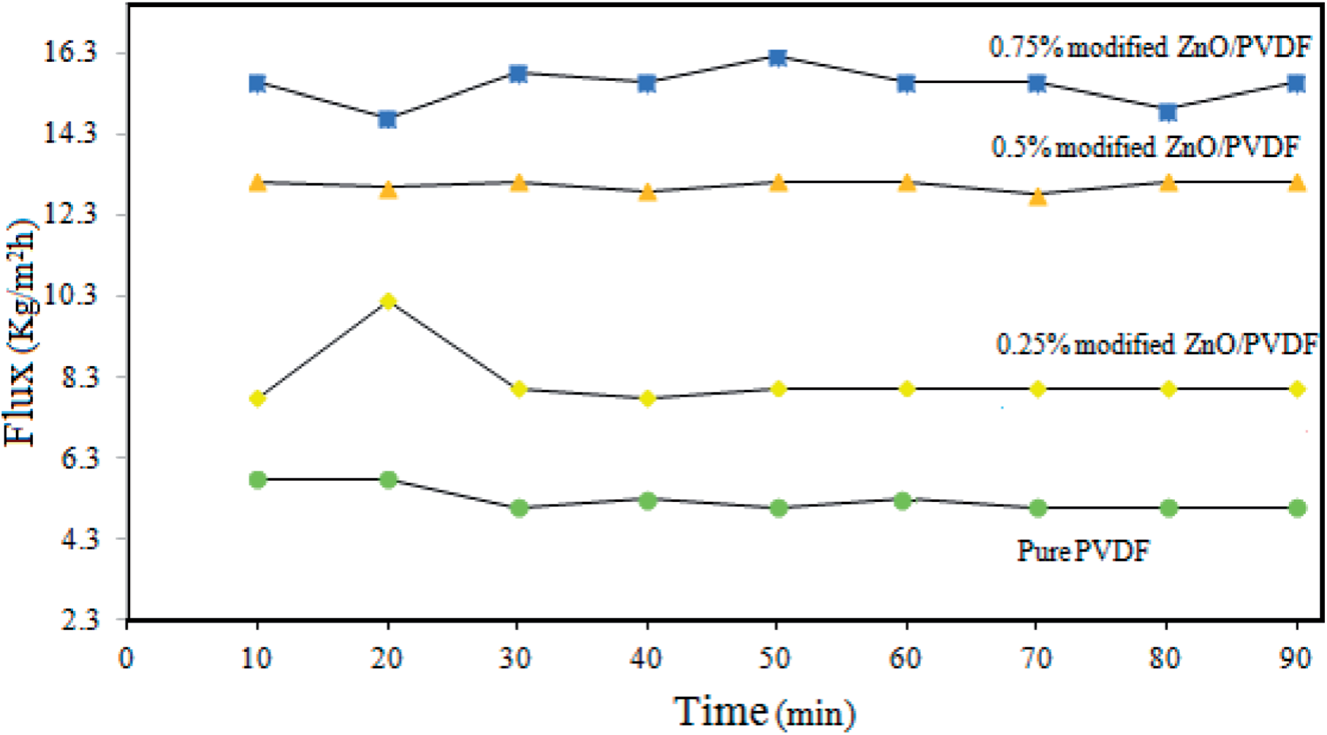
Effect of PS/ZnO concentration in the modified membrane on the DCMD performance.

The incorporation of the PS/ZnO hybrid nanocomposite into the PVDF membrane in addition to changes in the membrane properties led to the difference in the MD performance of the prepared membranes. The variations of flux *versus* time for the pure and 0.25, 0.5 and 0.75% PS/ZnO modified membranes are presented in Fig. 12. It was found that all membranes showed a constant flux curve and salt rejection was about 99.99% over 90 min. The flux obtained for the bare PVDF membrane was 7.89 kg m^−2^ h^−1^, which was the lowest value in compassion with the PS/ZnO hybrid nanocomposite-modified membranes. It can be observed from Fig. 12 that when the loading of PS/ZnO nanocomposite increased from 0 to 0.25%, the flux slightly increased. Further addition of PS/ZnO nanocomposite (0.5 and 0.75%) in the casting solution resulted in a sharp enhancement of the flux. A maximum value in the permeate flux (15.79 kg m^−2^ h^−1^) was observed for the PVDF membrane containing 0.75% PS/ZnO hybrid nanocomposite. As a result, the interplay between pore size and surface roughness can be considered as the main reason for the obtained flux. However, with higher concentration of nanocomposite, the pore size was more important than the surface roughness, which is in accordance with the FESEM and AFM results.

### 3.7. Long-term test

**Fig. 13:**
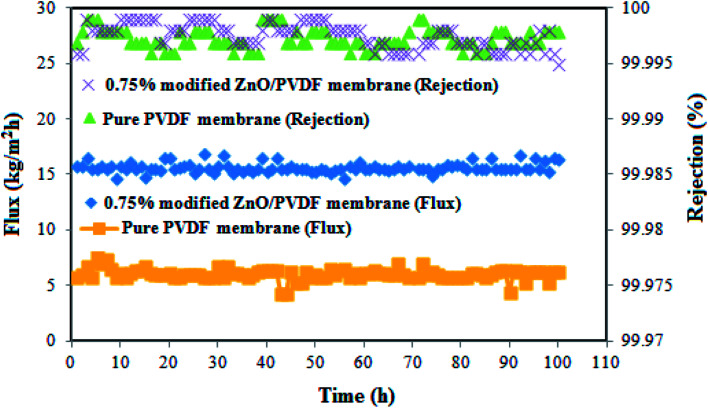
Variation of the membrane flux during 100 hours continuous desalination process.

Fig. 13 shows the flux and rejection obtained during 100 h of operation for the pure PVDF and 0.75% incorporated PS/ZnO nanocomposite membranes. The 0.75% PS/ZnO membrane was selected to apply for the long-term test owing to it having a higher flux than the other modified membranes in the distillation of saline solution. In this case, the DCMD experiments were carried out using 30 g L^−1^ NaCl salt solution; the temperatures of the hot feed and cold permeate were kept constant at 70 °C and 22 °C, respectively. The results indicated that both of the membranes exhibited rejection of about 99.99%. However, the modified membrane containing 0.75% PS/ZnO hybrid nanocomposite had admirable performance stability in comparison with the pure PVDF membrane because of its high porosity and appropriate LEP. It is believed that the PS/ZnO hybrid nanocomposite entrapped in the PVDF membranes can improve the flux of the PVDF membrane, which has great potential for desalination of saline solution in high concentrations for MD applications.

In order to compare the characteristics as well as the DCMD performance of different PVDF nanocomposite membranes with those of the membranes prepared in the current work, a comprehensive literature survey was done and the results are presented in Table 4. It seems that the DCMD performance of the present nanocomposite membranes is comparable with the modified PVDF membranes used so far for the MD process. For instance, Agbajea and coworkers^72^ are prepared the PVDF membranes by incorporating Fe_3_O_4_ nanoparticles into casting solution that obtained flux about 14 kg m^−2^ h^−1^. The permeate flux of the composite membrane prepared in this work was higher than those of PVDF membranes blended with Cloisite 15 (ref. 73) and clay^74^ though the inlet temperature and porosity were higher in comparison with those from our study. The current study suggests high MD performance for the nanocomposite membrane because of its high porosity, appropriate LEP values and especially the strong interaction between the PS/ZnO and PVDF matrix can facilitate water vapor molecule penetration through the micro pores and thereby increase the flux.

**Table 4:** Comparison of the maximum flux obtained in this study with literature reports for DCMD processes

Membrane	Feed solution		LEP (bar)	Porosity (%)	Flux (kg m^−2^ h^−1^)	Ref.
	NaCl concentration	Inlet temperature (°C)				
PVDF/SiO_2_/PDMS	3.5%	50	27	68	8.5	69
PVDF/fluorinated TiO_2_	5%	40	0.46	56	6.8	70
PVDF/CaCO_3_	3%	53	8.9	64.36	14.1	71
PVDF/Fe_3_O_4_	3.5%	60	2.2	58	14	72
PVDF-Cloisite 15 A®	Distilled water	90	0.9	83.03	7	73
PVDF/clay	3.5%	80	2	81	5.7	74
PS-ZnO/PVDF	3%	70	6	73	15.79	In this study

## 4. Conclusion

In the current work, nanocomposite membranes for MD application were successfully fabricated by loading different concentrations of PS/ZnO hybrid nanocomposite (0–0.75%) into the PVDF polymer matrix through phase inversion method. The PS/ZnO nanocomposite was synthesized *via* free radical polymerization of VTMS from vinyl groups grafted on the ZnO nanoparticle surface with styrene monomer. It was found that the morphology changes caused by incorporating the PS/ZnO nanocomposite into the PVDF membrane led to increased pore size and surface roughness, which favored mass transfer resistance reduction to vapor molecules through the membrane pores in the MD process. The obtained results showed that the porosity of modified membrane was increased to 73% by introducing the nanocomposite, although the LEPw values were reduced to some extent. In the end, during the DCMD experiment using 30 g L^−1^ salt solution as the feed at 70 °C, the PVDF membrane containing 0.75% PS/ZnO nanocomposite showed twice the flux and higher MD performance compared with the other nanocomposite membranes and the neat PVDF membrane.

## Conflicts of interest

There are no conflicts to declare.

## Supplementary Material
